# Intra-cardiac thrombus detection by electrocardiogram-gated cardiac computed tomography in hyperacute ischemic stroke

**DOI:** 10.3389/fneur.2026.1762455

**Published:** 2026-06-24

**Authors:** Daniel S. Green, Timmy Pham, Dennis J. Cordato, Longting Lin, Daniel Akrawi, Callan Gavaghan, Chilan Nguyen, Aaron Gaekwad, Alan McDougall, Carlos Garcia-Esperon, Anthony Kaplan, Christopher Blair, Melissa Leung, Mark W. Parsons

**Affiliations:** 1 South Western Sydney Clinical School, University of New South Wales, Sydney, NSW, Australia; 2 Ingham Institute for Applied Medical Research, Liverpool, NSW, Australia; 3 Department of Cardiology, Liverpool Hospital, Liverpool, NSW, Australia; 4 Department of Neurology, Liverpool Hospital, Liverpool, NSW, Australia; 5 School of Medicine and Public Health, University of Newcastle, Newcastle, NSW, Australia; 6 School of Medicine, Western Sydney University, Campbelltown, NSW, Australia; 7 Cardiology Department, Queen Elizabeth II Jubilee Hospital, Coopers Plains, QLD, Australia; 8 Neurology and Neurophysiology Department, Liverpool Hospital, Liverpool, NSW, Australia; 9 Department of Neurology, John Hunter Hospital, Newcastle, NSW, Australia; 10 Faculty of Medicine, University of Newcastle, Newcastle, NSW, Australia; 11 Hunter Medical Research Institute, Newcastle, NSW, Australia; 12 Department of Radiology, Liverpool Hospital, Liverpool, NSW, Australia

**Keywords:** acute, cardiac CT, echocardiogram, gated, ischemic stroke, thrombus, TTE

## Abstract

**Background:**

Traditional workup with transthoracic echocardiogram (TTE) has limited yield in the diagnosis of suspected cardioembolic stroke. The current study aimed to determine the diagnostic yield of hyperacute electrocardiogram (ECG)-gated cardiac computed tomography (CT) in comparison with TTE in the detection of intra-cardiac thrombus amongst patients with ischemic stroke within an ethnically and socioeconomically diverse population.

**Methods:**

A prospective observational analysis was undertaken of patients who had undergone cardiac CT with prospective ECG-gating as part of a hyperacute stroke imaging protocol at a single comprehensive stroke centre in Sydney, Australia between November 2022 and December 2024. Data related to demographics, clinical assessment, treatment and follow-up functional status were collected in addition to high-risk sources of cardio-aortic stroke.

**Results:**

A total of 117 patients underwent imaging with ECG-gated cardiac CT as part of their hyperacute stroke assessment. Of 69 (59%) patients with a final diagnosis of ischemic stroke, intra-cardiac thrombus was identified on cardiac CT in 18 patients (26%). Intra-cardiac thrombus was associated with a higher prevalence of premorbid hypertension (94% vs. 67%; *p* = 0.03), premorbid heart failure (33% vs. 2%; *p* < 0.001), premorbid atrial fibrillation or atrial flutter (56% vs. 24%; *p* = 0.01) and intracranial arterial occlusion (83% vs. 49%; *p* = 0.01). Amongst patients who underwent both cardiac CT and TTE (*n* = 49; 71%), intra-cardiac thrombus was more likely to be identified on cardiac CT than TTE (29% vs. 2%; *p* < 0.001). In an unadjusted analysis, more patients had poor functional outcome (modified Rankin scale 4–6) at 3 months if intra-cardiac thrombus was present on cardiac CT (67% vs. 28%; *p* = 0.03).

**Conclusion:**

ECG-gated cardiac CT identified intra-cardiac thrombus in 26% of patients who had a final diagnosis of ischemic stroke. The diagnostic yield of intra-cardiac thrombus detection was substantially higher with cardiac CT compared to TTE. Cardiac CT should be considered as part of hyperacute stroke imaging protocols. The unadjusted analysis on functional outcomes is hypothesis generating, limited by imprecision from a small sample size; further investigation requires a larger cohort with multivariable adjustment. Further studies in the hyperacute setting are required to explore benefits in different populations and with alternative imaging acquisition protocols.

## 1 Introduction

Ischemic stroke has a range of underlying etiologies, of which cardioembolic sources account for almost one third (1). In comparison to other causes of stroke, cardioembolic stroke is associated with a high rate of recurrence, increased severity and worse fatality rates (1–3). Cardiac sources of embolism are also postulated to be one of the underlying mechanisms of cryptogenic stroke, an entity that accounts for approximately 25% of ischemic strokes (4). Typical workup to assess for a cardioembolic source is with transthoracic echocardiography (TTE), although concern exists regarding yield, with one study demonstrating it changed management in 5% of patients (5). Transesophageal echocardiography (TEE) has been demonstrated to have superior yield, but limitations to timely access and other logistical challenges are barriers in its usage (6).

Cardiac computed tomography (CT) has become increasingly evaluated as a tool to identify high-risk embolic sources in patients with ischemic stroke (7–9). In comparison with traditional echocardiography, cardiac CT has been shown to have a sensitivity and specificity of approximately 98 and 96% for detecting intra-cardiac thrombus (8). More recently, cardiac CT has become incorporated in some centres as part of the hyperacute diagnostic workup of ischemic stroke and has been shown to identify high-risk cardio-aortic source of embolism twice as often in comparison with TTE (10). Hyperacute assessment with cardiac CT may offer earlier detection and treatment of intra-cardiac pathology, which can be time-critical, compared with image acquisition at a later stage, such as with a standard cardiac CT or traditional echocardiography, which typically does not occur at the time of initial stroke imaging. Additionally, cardiac CT is non-invasive and may be more accessible in comparison with TEE, which, even in the setting of cryptogenic stroke, has been shown to be undertaken infrequently (11).

Existing data related to cardiac CT in the hyperacute setting largely pertain to single-centre studies, which may affect extrapolation of results (10, 12–16). Electrocardiogram (ECG)-gating in addition to delayed-phase imaging have been shown to further optimise specificity and sensitivity for intra-cardiac thrombus detection although data in the hyperacute setting are limited (8, 10, 12, 17). In the recent DAYLIGHT study, cardiac imaging occurred as part of an extension of cranio-cervical CT angiography as opposed to a separate scan protocol, and the role of ECG-gating or delayed-phase imaging was highlighted as an area for future investigation (18).

In the setting of the heterogeneity of existing data, we aimed to compare the diagnostic utility of ECG-gated cardiac CT during hyperacute stroke assessment with TTE in the identification of intra-cardiac thrombus within an ethnically and socioeconomically diverse population. The secondary objective was to assess the utility of cardiac CT with respect to other high-risk cardio-aortic sources of embolism, along with treatment implications for these findings, and clinical outcomes.

## 2 Materials and methods

### 2.1 Study design

A prospective observational study was undertaken of the South Western Sydney Stroke Registry. This registry is a prospectively collected observational database related to patients undergoing assessment for stroke syndromes within hospitals in the South Western Sydney Local Health District.

### 2.2 Patient selection

All patients included were from a single, comprehensive stroke centre where ECG-gated cardiac CT was part of the hospital’s hyperacute stroke imaging protocol in those over 18 years of age with a stroke syndrome potentially eligible for reperfusion therapy. All patients who had undergone cardiac CT as part of their hyperacute stroke assessment, which was performed by the acute stroke team, and had a final confirmed diagnosis of ischemic stroke were included from between November 2022 and December 2024. Patients also underwent a non-contrast CT brain, CT angiogram (CTA) of the aortic arch to Circle of Willis and CT perfusion study as per hospital protocol.

### 2.3 Ethics approval

The South Western Sydney Stroke Registry was approved by the South Western Sydney Local Health District Human Research Ethics Committee (ETH00096).

### 2.4 Imaging protocol

A 256-slice CT scanner (GE Revolution™ Apex Platform) was utilised for imaging acquisition and located adjacent to the hospital’s emergency department. A non-contrast CT brain was initially undertaken using a fixed 120 kVp technique. A first dose of iodinated contrast (50 mL of Omnipaque 350) was then administered to facilitate a CT cerebral perfusion study, followed by a 50 mL normal saline flush. The scan range or *z*-axis for the perfusion was 120 mm and acquisition parameters were 80 kVp and 115 mAs. Two to three minutes after this first contrast dose, a second dose of iodinated contrast (50 mL of Omnipaque 350) was administered followed by a 50 mL normal saline flush. A CTA of the aortic arch to the Circle of Willis was then obtained using bolus tracking and with a tube voltage between 80 and 120 kVp.

Approximately 3 min after completion of the CT angiogram, a third dose of iodinated contrast (70 mL of Omnipaque 350) was administered using a split bolus technique followed by a 50 mL normal saline flush. A prospectively gated cardiac CT scan was then triggered using a bolus tracking technique with a trigger set at the ascending aorta. Imaging was obtained during diastole. If the heart rate exceeded 71 bpm, imaging during systole was also obtained to improve yield due to motion artefact. The patient’s arms were positioned during the cardiac CT scan adjacent to their head if tolerated and assistance was provided if required. Cardiac CT acquisition was estimated to have been completed by approximately 5 min after the end of the CT angiogram acquisition. The combination of imaging acquired from cardiac CT and CT angiogram allowed for visualisation of the ascending aorta and aortic arch. See Supplementary Figure 1.

Secondary reconstruction of systolic imaging was undertaken using SnapShot Freeze, a GE software, in conjunction with the GE Advanced Workstation (AW) server. The tube voltage was 80–120 kV. Axial images for the cardiac CT were reconstructed with a section thickness of 0.625 mm and an increment of 0.625 mm, with a standard kernel with true fidelity. Pharmacological agents, including nitrates and beta blockers, were not administered in view of the time-critical nature of imaging acquisition and to avoid hypotension in the setting of acute stroke.

### 2.5 Data collection

Data were retrieved from the database with respect to baseline demographics and medical comorbidities, acute stroke assessment, treatments administered, new diagnoses of atrial fibrillation or atrial flutter (AF) and three-month modified Rankin scale (mRS) scores. High-risk cardio-aortic sources of embolism were defined by study authors (D. G., M. L., M. P., and D. C.) and were informed by existing literature (10, 19–21). These high-risk sources included: intra-cardiac thrombus; papillary fibroelastoma; signs of endocarditis; prosthetic valve pannus or thrombus; Stanford classification type A acute aortic dissection; atrial myxoma; akinetic segments of the left ventricle; left ventricular (LV) ejection fraction (EF) ≤ 35%; and complex aortic atherosclerotic plaque proximal to the suspected affected vascular territory, which was defined as atheroma >4 mm, ulceration or the presence of mobile thrombus.

Cardiac CT images were reviewed by two study authors (D. G. and D. A.), under supervision from a study author (M. L.). Variables were also extracted from cardiac CT reports produced by a radiologist with expertise in cardiac CT (A. K.). Reports and images, where required, of TTE and TEE studies were reviewed by two study authors (D. G. and C. N.) under supervision from a study author (M. L.).

TTE and TEE studies were only included in the analysis if undertaken at the local hospital as part of the admission or up until 3 months after discharge. Readers of cardiac CT were not blinded to clinical data. Readers of TTE and TEE were not blinded to results of cardiac CT. Intra-cardiac thrombus was defined on cardiac CT as a low-attenuated mass, typically with a filling defect of < 100 Hounsfield Units, whereas slow flow was defined as an inhomogeneous defect, typically ≥ 100 Hounsfield Units (10). Etiologies of stroke were determined by two study authors (D. G. and D. C.) in accordance with the Trial of Org 10,172 in Acute Stroke Treatment (TOAST) subtype classification system and criteria proposed for embolic strokes of undetermined source (ESUS) (4, 22).

### 2.6 Primary outcome

The primary outcome was the identification of intra-cardiac thrombus on cardiac CT in comparison with TTE.

### 2.7 Secondary outcomes

Secondary outcomes were undertaken where possible to determine: the diagnostic yield of intra-cardiac thrombus on cardiac CT versus TEE; the diagnostic yield of cardiac CT, TTE and TEE in identifying other high-risk cardio-aortic sources of embolism; the effect of intravenous thrombolysis on intra-cardiac thrombus detection by echocardiography; the association of intra-cardiac thrombus on cardiac CT with timing and prescription of anticoagulation commencement or recommencement; and the effect of intra-cardiac thrombus on cardiac CT with respect to three-month mRS scores.

### 2.8 Statistical analyses

Frequencies and percentages were reported for categorical outcomes. Median and IQR, expressed as Q1–Q3, or mean with standard deviation was reported for quantitative variables as appropriate. The Shapiro–Wilk test was utilised to determine the normality of distribution of quantitative variables. A Fisher exact test, *χ*^2^ test, Mann–Whitney test and *t*-test were utilised as appropriate for categorical and quantitative data. A two-sided McNemar test was undertaken to assess the primary outcome. A significance level of 0.05 was used for all tests. Statistical analyses were performed using STATA software, version 18.5 SE-Standard Edition.

## 3 Results

A total of 117 patients underwent imaging with ECG-gated cardiac CT as part of their hyperacute stroke assessment. A final diagnosis of TIA was made in 15 (13%) patients, whilst stroke mimics were diagnosed in 33 (28%) patients. The remaining 69 (59%) patients had a final diagnosis of ischemic stroke and were included in the analysis.

The mean age of patients was 72 ± 12.7 years and 33 (48%) were female. Nineteen (28%) patients were born in Australia. Other countries of birth within Oceania included Fiji and New Zealand, each where three patients were born. The most common continent of birth was Asia (*n* = 26; 38%). See Table 1. Intra-cardiac thrombus was identified on cardiac CT in 18 patients (26%). Slow flow within the LAA was present on cardiac CT in an additional four patients (6%). Imaging of the left atrium was of sub-optimal diagnostic utility in one included study, however visualisation of the LAA was sufficient and demonstrated slow flow.

**Table 1:** Characteristics of patients with ischemic stroke, stratified according to the presence of intra-cardiac thrombus on cardiac CT.

Variable	Intra-cardiac thrombus detected by cardiac CT			
	No	Yes	Total	*p* value
	*n* = 51	*n* = 18	*n* = 69	
Sex				
Male	28 (55%)	8 (44%)	36 (52%)	0.445
Female	23 (45%)	10 (56%)	33 (48%)	
Age	72 (12.3)	74 (14.0)	72 (12.7)	0.511
Born in Australia	14 (27%)	5 (28%)	19 (28%)	0.979
Continent of birth				
Africa	1 (2%)	2 (11%)	3 (4%)	0.017
Asia	24 (47%)	2 (11%)	26 (38%)	
Europe	9 (18%)	6 (33%)	15 (22%)	
Oceania	17 (33%)	8 (44%)	25 (36%)	
Premorbid mRS	0 (0–2)	1 (0–2)	1 (0–2)	0.637
Premorbid hypertension	34 (67%)	17 (94%)	51 (74%)	0.027
Premorbid diabetes	14 (27%)	8 (44%)	22 (32%)	0.184
Premorbid dyslipidemia	29 (57%)	12 (67%)	41 (59%)	0.466
Premorbid IHD	11 (22%)	5 (28%)	16 (23%)	0.592
Premorbid HF	1 (2%)	6 (33%)	7 (10%)	<0.001
Premorbid previous stroke	12 (24%)	1 (6%)	13 (19%)	0.160
Premorbid previous TIA	9 (18%)	2 (11%)	11 (16%)	0.715
Premorbid active malignancy	2 (4%)	1 (6%)	3 (4%)	1.000
Premorbid venous thrombosis (i.e. PE, DVT and/or CVT)	1 (2%)	2 (11%)	3 (4%)	0.165
Diagnosis (past or new) of a hypercoagulable state	0 (0%)	0 (0%)	0 (0%)	
Premorbid smoking status				
Never smoked	36 (73%)	14 (78%)	50 (75%)	0.905
Current or within past 6 months	5 (10%)	2 (11%)	7 (10%)	
Prior to 6 months ago	8 (16%)	2 (11%)	10 (15%)	
Premorbid antiplatelet therapy				
Nil	28 (56%)	10 (56%)	38 (56%)	0.871
Aspirin	14 (28%)	6 (33%)	20 (29%)	
Clopidogrel	5 (10%)	2 (11%)	7 (10%)	
Aspirin and clopidogrel	3 (6%)	0 (0%)	3 (4%)	
Premorbid anticoagulation therapy				
Nil	41 (82%)	11 (61%)	52 (76%)	0.070
Apixaban	5 (10%)	2 (11%)	7 (10%)	
Rivaroxaban	3 (6%)	5 (28%)	8 (12%)	
Dabigatran	1 (2%)	0 (0%)	1 (1%)	
AF—premorbid diagnosis	12 (24%)	10 (56%)	22 (32%)	0.012
AF—new diagnosis during admission	7 (14%)	4 (22%)	11 (16%)	0.460
NIHSS on presentation	8 (4–15)	15 (4–20)	9 (4–16)	0.204
Intracranial occlusion	25 (49%)	15 (83%)	40 (58%)	0.011
Extracranial occlusion	5 (10%)	1 (6%)	6 (9%)	1.000
Occlusion site				
Anterior circulation	21 (81%)	13 (87%)	34 (83%)	1.000
Posterior circulation	4 (15%)	2 (13%)	6 (15%)	
Both	1 (4%)	0 (0%)	1 (2%)	
Perfusion lesion—core	1 (0–12)	5 (1–38)	1 (0–13)	0.077
Perfusion lesion—penumbra	19 (0–73)	44 (8–90)	26 (0–76)	0.081
Perfusion lesion—total	22 (0–87)	64 (8–131)	31 (0–90)	0.093
Thrombolysis	11 (22%)	8 (44%)	19 (28%)	0.062
Endovascular thrombectomy	13 (25%)	8 (44%)	21 (30%)	0.133
Cardiac CT DLP (mGy*cm)	382 (312–443)	426 (379–518)	401 (318–481)	0.209

### 3.1 Intra-cardiac thrombus

Intra-cardiac thrombus was identified in the LAA on cardiac CT in 17 (25%) patients and in the left ventricle in one (1%) patient. Intra-cardiac thrombus on cardiac CT was significantly associated with a higher prevalence of premorbid hypertension (94% vs. 67%; *p = 0.03*), premorbid heart failure (33% vs. 2%; *p < 0.001*) and premorbid AF (56% vs. 24%; *p = 0.01*). Intracranial arterial occlusions, defined as occlusions affecting either large or medium vessels, were more likely in the presence of intra-cardiac thrombus (83% vs. 49%; *p = 0.01*).

Amongst the 49 (71%) patients who underwent both cardiac CT and TTE, intra-cardiac thrombus was more likely to be identified on cardiac CT (29%, 14/49) than TTE (2%, 1/49) (RR 14; 95% CI 2.12–92.55, *p* < 0.001, exact McNemar). See Table 2. Amongst patients with thrombus on cardiac CT but not on TTE, the median time until TTE acquisition from index stroke was 4.7 days (IQR 1.9–5.1). Amongst the eight (12%) patients who underwent both cardiac CT and TEE, intra-cardiac thrombus was identified on cardiac CT in three (38%) patients versus one (13%) patient with TEE, although this difference was not significant (*p* = 0.50; *exact McNemar*).

**Table 2:** The frequency of ischemic stroke patients with and without intra-cardiac thrombus on cardiac CT and TTE.

Variable	TTE—thrombus	TTE—no thrombus
Cardiac CT—thrombus	1	13
Cardiac CT—no thrombus	0	35

Intra-cardiac thrombus was identified in one (2%, 1/49) patient who underwent TTE and one (13%, 1/8) further patient who underwent TEE. In each of these two cases, thrombus was identified in the LAA. Amongst patients with intra-cardiac thrombus identified on cardiac CT, three underwent imaging with TEE. In each of these cases, thrombus was found in the LAA and was only identified in one TEE study (33%, 1/3). There were no cases where TTE or TEE identified intra-cardiac thrombus that was not also identified on cardiac CT.

Regarding other high-risk cardio-aortic sources of embolism, cardiac CT identified complex aortic arch atheroma in seven (10%) patients. An akinetic LV segment was identified in eight (16%, 8/49) patients who underwent TTE and one (13%, 1/8) patient who underwent TEE. An LVEF ≤35% was identified in six (12%, 6/49) patients who underwent TTE and one (13%, 1/8) patient who underwent TEE. See Table 3.

**Table 3:** Frequency of high-risk cardio-aortic sources of embolism in patients with ischemic stroke, stratified by imaging modality.

Source of embolism	Cardiac CT (*n* = 69)	TTE (*n* = 49)	TEE (*n* = 8)
Akinetic LV segment	N/A	8 (16%)	1 (13%)
Aortic dissection	0	0	0
Complex arch atheroma	7 (10%)	N/A	N/A
Endocarditis	0	0	0
Intra-cardiac thrombus	18 (26%)	1 (2%)	1 (13%)
LVEF ≤ 35%	N/A	6 (12%)	1 (13%)
Myxoma	0	0	0
Papillary fibroelastoma	0	0	0
Prosthetic valve pannus/thrombus	0	0	0

An assessment was made as to any potential confounding of echocardiography results by thrombolysis in patients who had intra-cardiac thrombus detected on cardiac CT. Of the 15 patients with intra-cardiac thrombus on cardiac CT who underwent either TTE or TEE, seven (47%, 7/15) patients underwent thrombolysis, none of whom subsequently had evidence of thrombus on echocardiography. Of the eight (53%, 8/15) patients who did not receive thrombolysis, two (25%, 2/8) patients had evidence of thrombus on subsequent echocardiography. This difference was not statistically significant (*p* = 0.47; Fisher exact). A *post-hoc* analysis found no difference in the median time to initial echocardiography assessment amongst those who received thrombolysis versus those who did not [4.9 days vs. 3.1 days; *p* = 0.35; Kruskal–Wallis (i.e. Mann–Whitney test/Wilcoxon rank-sum test)].

Fourteen (78%, 14/18) patients with intracardiac thrombus on cardiac CT were diagnosed with AF by the end of hospital admission, of whom 10 (71%, 10/14) had a premorbid diagnosis of AF. Of the four (22%, 4/18) patients who were not diagnosed with AF by the end of hospital admission, thrombus was identified in the LAA in three patients and left ventricle in one patient. Each of the four patients had a TTE undertaken, none of which demonstrated intra-cardiac thrombus; Definity ultrasound contrast agent was utilised as part of the TTE study for the patient with the LV thrombus identified on cardiac CT to improve LV cavity opacification and endocardial border delineation. One TEE study was undertaken in this cohort of four patients, which did not identify thrombus in the LAA that had been seen on cardiac CT. Thrombolysis had been administered to two of the four patients: one patient who had LAA thrombus on cardiac CT with a subsequent negative TEE; and one patient who had LV thrombus on cardiac CT with a subsequent negative TTE utilising Definity contrast.

Amongst patients with a premorbid history of AF, the median time to commence or recommence anticoagulation in those with and without intra-cardiac thrombus on cardiac CT was not significantly different (4.9 days vs. 3.4 days respectively; *p = 0.50; two-sample Wilcoxon rank-sum test exact probability*).

Amongst the cohort of patients without a diagnosis of AF by the end of hospital admission, anticoagulation was more likely to be prescribed if intra-cardiac thrombus was present on cardiac CT (100%, 4/4) compared to if no intra-cardiac thrombus was present on cardiac CT (14%, 4/29) (*p* = 0.002; *Fisher exact*). When further selecting for those who were not already prescribed anticoagulation prior to admission, anticoagulation was more likely to be prescribed if intra-cardiac thrombus was present on cardiac CT (100%, 3/3) compared to when no intra-cardiac thrombus was present on cardiac CT (4%, 1/26) (*p* = 0.001; *Fisher exact*).

Two (3%) patients had stroke etiology reclassified with the additional information provided by cardiac CT. In both cases, the etiology was changed to ‘two or more potential causes’. Twenty (29%) patients did not undergo assessment with transthoracic echocardiography and so were not eligible for assessment of an underlying ESUS diagnosis. See Table 4.

**Table 4:** The frequency of stroke etiologies when incorporating the additional information provided by cardiac CT.

Stroke etiology	Study cohort (*n* = 69)
Large artery atherosclerosis	4 (6%)
Cardioembolism	26 (38%)
Small vessel occlusion/disease	8 (12%)
Stroke of other determined etiology	2 (3%)
Unknown	
Two or more potential causes	13 (19%)
Incomplete evaluation	10 (14%)
Negative evaluation	6 (9%)
ESUS diagnosis	6 (9%)

**Figure 1:**
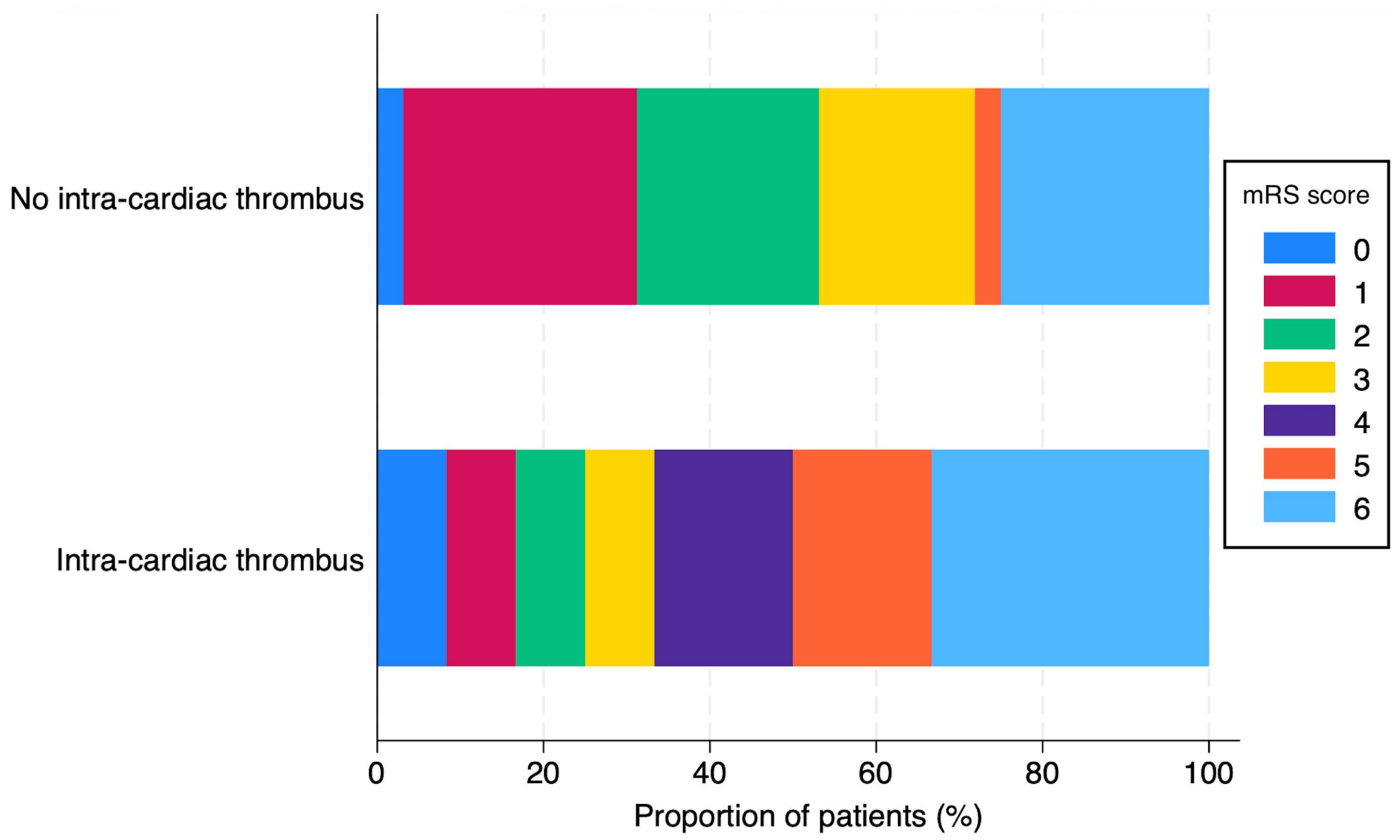
Modified Rankin Scale (mRS) scores at 3 months in patients with ischemic stroke, stratified according to the presence of intra-cardiac thrombus on cardiac CT.

In an unadjusted analysis, there were more patients with poor outcome (mRS 4–6) at three months if intra-cardiac thrombus was present on cardiac CT (67%, 8/12) compared to if intra-cardiac thrombus was not present on cardiac CT (28%, 9/32) (OR 5.11; CI 1.23–21.28; *p* = 0.03). Multivariable adjustment was not feasible in the context of the small number of observations (*n* = 44), and so this analysis is reported as exploratory. The distribution of mRS outcomes at 3 months is detailed in Figure 1.

## 4 Discussion

We identified intra-cardiac thrombus in 26% of patients with ischemic stroke who had undergone hyperacute ECG-gated cardiac CT. This diagnostic yield was notably higher than in comparable studies, where yields ranged from 3.9–13.5% (10, 12–16, 23, 24). There are several potential explanations for this higher rate of intra-cardiac thrombus detection. We found that premorbid AF was associated with intra-cardiac thrombus detection, in keeping with the widely acknowledged fact that AF is a risk factor for intra-cardiac thrombus. Perhaps more accurately, both AF and intra-cardiac thrombus may be regarded as downstream markers of underlying atrial myopathy (25, 26). The prevalence of premorbid AF amongst all patients in the current study was 32%, which was higher than the 17.1% reported in a recent study with a similar imaging protocol (10). The disparity in the prevalence of premorbid AF may have contributed to the higher diagnostic yield of intra-cardiac thrombus in our study.

Premorbid hypertension was associated with the presence of intra-cardiac thrombus on cardiac CT. A plausible mechanism may also be of an underlying atrial myopathy, which is commonly related to hypertension, predisposing to LA thrombus formation (27). The prevalence of premorbid hypertension we observed was higher than that seen in a similar study (74% vs. 46.2% respectively), a finding that may have contributed to the increased diagnostic yield for intra-cardiac thrombus (10). Challenging this interpretation, however, are two recent studies that did not show an association between hypertension and intra-cardiac thrombus (albeit with much lower rates of LAA thrombus) (13, 24).

Disparities in diagnostic yield of intra-cardiac thrombus with other studies may also partly be due to differences in study designs. Patients enrolled in some other hyperacute studies have included those with suspected cardio-embolic stroke, suspected large vessel occlusion or stenosis, and those with clinical stroke syndromes (12, 13, 24, 28, 29). Cardiac CT was considered at the institution from the current study in patients with a stroke syndrome eligible for reperfusion therapy, a criterion that has also been published elsewhere (10). It is possible that this requirement increased the proportion of cases with a higher stroke severity, a clinical feature associated with cardioembolic stroke, and so therefore optimised the diagnostic yield of cardiac CT (2).

The demographics of the underlying population in the current study may also have contributed to the relatively higher yield of intra-cardiac thrombus. South West Sydney is a culturally and linguistically diverse (CALD) community. People from CALD backgrounds can experience a range of barriers at an individual, community and systems levels with respect to health services utilisation (30). In South West Sydney, most residents reside in local suburbs that are well recognised as having higher levels of socioeconomic disadvantage, which, is linked to increased risk factors for disease and reduced utility of health prevention services (31). In 2017, South West Sydney was also home to almost two thirds of humanitarian entrants and refugees arriving in New South Wales, a population that is particularly affected by suboptimal health literacy and economic hardship (31). Such factors may have contributed to the higher prevalence of comorbidities, such as AF and hypertension, in the study population. Additionally, management of these conditions may also have been sub-optimal, however this was not specifically measured in our study.

The specific imaging protocol utilised at the institution from this study likely also contributed to the relatively high diagnostic yield. The utilisation of ECG-gating may have optimised diagnostic yield by increasing temporal resolution and reducing the occurrence of motion artefact (32). Acquisition of cardiac imaging was obtained in the current study following dedicated contrast boluses for the CT perfusion, CT angiogram and CT cardiac scans. In this way, simulation of both ‘early’ and ‘delayed’ cardiac imaging was produced in one phase rather than from two separate acquisitions. Delayed phase imaging may help to differentiate intra-cardiac thrombus from circulatory stasis by allowing more time for the LAA to fill, which may be of particular importance in AF (33). A similar contrast protocol with delayed imaging, although with retrospective gating, was incorporated by Austein et al. (28), whilst Rinkel et al. (10) stated that their prospective ECG-gated protocol ‘mimicked’ a delayed scan, also with a similar contrast regimen. Amongst published imaging protocols, time intervals used to denote ‘delay’ vary considerably, with some using only 30 s, and others up to 6 min (7, 15, 16, 34, 35). In a meta-analysis comparing cardiac CT and TEE, heterogeneity between studies incorporating delayed imaging protocols was reduced when the interval between contrast injection and image capture was more than 1 min (17).

In comparison with some other cardiac CT protocols, the contrast regimen, delayed phase imaging and separate acquisition sequence required for ECG-gating at the institution from this study may be more logistically challenging and require additional technical expertise from radiographers. The median dose-length product from cardiac CT at the current institution was 401 mGy*cm, which is equivalent to 10.4 mSv when multiplied by the conversion factor *k* of 0.026 mSv*mGy^−1^ cm^−1^, and is regarded as a low dose of radiation (36, 37). This radiation dose was higher in comparison to a similar recent study, and is likely related to additional acquisition of imaging during systole to optimise image quality, particularly with respect to coronary artery assessment (10).

The disparity in yield between cardiac CT and TTE in our study may be related to modality limitations, such as suboptimal visualisation of the LAA with TTE. It may also reflect the longer time interval to obtain TTE, which theoretically increases chance of thrombus dissolution, a process that has been observed to occur following thrombolysis over hours to days (38–40). One patient in the current study who received thrombolysis was found to have a LV thrombus on cardiac CT but not on TTE despite the use of Definity contrast; this scan did not occur until 58 days after the stroke due to logistical reasons. In our study, we allowed for TTE up to 3 months following cardiac CT, which may have affected diagnostic yield. Further supporting the possibility of thrombus dissolution were the results of the three TEE investigations undertaken on patients with LAA thrombus in the current study: one patient, who did not receive thrombolysis, had evidence of thrombus on TEE undertaken approximately 4 days after cardiac CT; the other two, both of whom received thrombolysis, had no evidence of thrombus on TEE performed one and 7 days after cardiac CT. Intra-cardiac thrombus dissolution in the absence of thrombolysis has also been described in the literature, further emphasising the potential importance of its timely identification via hyperacute cardiac CT (28). The potential for thrombus dissolution limits the ability to make conclusions as to true diagnostic superiority of cardiac CT.

Amongst patients with intra-cardiac thrombus on cardiac CT in the present study, a new diagnosis of AF was made in four patients. Of additional interest were four patients in the present study who had intra-cardiac thrombus identified on cardiac CT, but no diagnosis of AF by the end of their hospital admission. In all four patients, accompanying echocardiography did not identify intracardiac thrombus, including one patient who had a TEE. A change in management, specifically the commencement of anticoagulation, occurred in three of these patients, representing 17% of those with intra-cardiac thrombus on cardiac CT. In each of the three cases, thrombus was isolated to the LAA and there was no premorbid requirement for anticoagulation. With respect to timing of anticoagulation following acquisition of cardiac CT: one patient with a small ischemic stroke commenced rivaroxaban on day three; one patient with a small ischemic stroke commenced therapeutic enoxaparin on day four; and one patient, who did not have MRI imaging available to further characterise the infarct, commenced apixaban on day four.

The finding of intra-cardiac thrombus on cardiac CT has the potential to assist clinicians in certain circumstances where clinical decision making as to anticoagulation requires careful risk and benefit analysis. Furthermore, even in the setting of known AF, earlier identification of intra-cardiac thrombus may also change management. In a survey of 402 stroke neurologists from 64 countries, the presence of a LAA thrombus in the setting of AF would: prompt most responders to undertake earlier anticoagulation due to increased embolic risk; increase suspicion amongst most responders of AF as an underlying cause in situations in the presence of a competing cause; and prompt most responders to consider an alternative preventative option, such as LAA closure (41).

Although not the primary focus of this study, the finding of slow flow in four (6%) patients may also reflect atrial dysfunction. The term ‘slow flow’ is often used as a proxy on cardiac CT for the echocardiographic phenomenon of spontaneous echocardiographic contrast (SEC) or LAA flow stasis, a strong risk factor for LAA thrombus formation (33, 42). Spontaneous echocardiographic contrast often occurs in AF but has been reported in patients with dilated left atria in sinus rhythm (43). The appearance of slow flow on cardiac CT occurs in the setting of incomplete mixing of contrast and blood and has been variably defined as resolution of contrast filling defects on delayed imaging but also based on appendage filling defects of ≥100 Hounsfield Units (10, 33, 44). *Post-hoc* analysis showed that all four patients with slow flow had a large or medium vessel intracranial occlusion, and three had a diagnosis of AF by the end of admission. In a recent study, at two-year follow-up, patients with LAA slow flow were found to have a similar rate of newly diagnosed AF as patients with LAA thrombus, underscoring the importance of prolonged rhythm monitoring in patients with slow flow (44). In the current study, inclusion of both slow flow and intra-cardiac thrombus within one category resulted in a diagnostic yield of 32% (22/69), of whom 23% (5/22) had no diagnosis of AF.

The potential for filling defects secondary to slow flow, thrombus and motion artefact, draws attention to the challenge of using a simple dichotomy to model the presence or absence of thrombus. It is possible that a gradual transition exists between filling states, which raises the question as to whether alternative methods of analysis could be used without creating explicit ‘cut-offs’. Fuzzy set theory is a mathematical framework that allows for grades of membership as opposed to establishing rigid cut-offs, and so may represent a future approach to model the graduality of filling defects (45). A recent study in another field of research demonstrated that fuzzy set theory, used in conjunction with two other mathematical models termed ‘Tversky similarity’ and a ‘Mamdani-type fuzzy inference system’, resulted in improved modelling of gradual transitions and spectral ambiguity in comparison to an alternative method that incorporated ‘crisp’ classifications (46).

In an exploratory, unadjusted analysis, a higher rate of moderate to severe disability (mRS 4–6) at 3 months was observed in those who had intra-cardiac thrombus identified on cardiac CT. This finding should be considered hypothesis-generating, however, in the absence of multivariable adjustment. There is also high susceptibility to residual confounding given the impression of the estimate, attributable to a small sample size and wide confidence interval.

Several limitations may have confounded the interpretation of the results. Firstly, the retrospective nature of the analysis, clinical suitability for cardiac CT and logistical challenges, such as radiographer or scanner availability, may have affected the ability to obtain consecutive scans in those otherwise eligible and introduced a selection bias. Second, the total number of patients with a suspected stroke throughout the entire study period was not ascertained, including those eligible for reperfusion therapy and who did not receive cardiac CT, which may have further introduced a selection bias. Third, it is possible that the lack of requirement for rate control during cardiac CT acquisition may have increased the number of cases with slow flow in the LAA. Fourth, incomplete echocardiography in the sample size of 69 patients limits comparisons with cardiac CT but also reflects a real-world context. Data were not present for TTE studies in 20 (29%) patients, for which there may be several reasons: clinicians may have determined TTE studies were not required based on suspected stroke etiology or from information provided by cardiac CT; some studies may have occurred at an external institution; or some patients’ conditions deteriorated in hospital such that TTE was unlikely to change clinical management. Only eight (12%) patients underwent TEE, which is considered the reference benchmark in identification of LAA thrombus. Our study was underpowered to compare cardiac CT and TEE, so this comparison was undertaken only as an exploratory analysis. Fifth, as a single center study with a relatively high proportion of AF and hypertension, the generalizability of these results to a broader stroke population may be limited. These risk factors may have contributed to an increased diagnostic yield of intra-cardiac thrombus. At other centres, cardiac CT protocols may not utilise ECG-gating or delayed sequence imaging, which may reduce the diagnostic yield of thrombus. Underlying differences in patient characteristics may have also contributed to the observation from an unadjusted analysis that more patients had poor functional outcome in the presence of intra-cardiac thrombus.

In conclusion, ECG-gated cardiac CT, undertaken as part of a hyperacute stroke imaging protocol, identified intra-cardiac thrombus in a high proportion (26%) of patients diagnosed with ischemic stroke. There was a much higher diagnostic yield of intra-cardiac thrombus identified by cardiac CT in comparison with routine TTE. Management with respect to anticoagulation was changed in 17% of patients who had intra-cardiac thrombus detected on cardiac CT. The unadjusted, exploratory analysis on functional outcomes is hypothesis generating, limited by imprecision in the context of a small sample size; further investigation would require a larger cohort with multivariable adjustment. The results of this study add to the growing evidence for the utility of cardiac CT as part of hyperacute stroke imaging protocols. Further studies in the hyperacute setting will be required to analyse benefits of cardiac CT in different populations and with alternative imaging acquisition protocols.

## Data Availability

The datasets presented in this article are not readily available because they are contained as part of a local hospital registry. Requests to access the datasets should be directed to the Data Custodian – Stroke Registry at the following email (SWSLHD-StrokeRegistry@health.nsw.gov.au).

## Supplementary material

SUPPLEMENTARY FIGURE 1 Schematic diagram outlining order of imaging and time intervals between end of CT angiogram, ECG-gated cardiac CT and echocardiography. * = approximation.

## References

[1] Kolominsky-Rabas PL Weber M Gefeller O Neundoerfer B Heuschmann PU. Epidemiology of ischemic stroke subtypes according to TOAST criteria: incidence, recurrence, and long-term survival in ischemic stroke subtypes: a population-based study. Stroke. (2001) 32:2735–40. doi: 10.1161/hs1201.100209, 11739965

[2] O'Carroll CB Barrett KM. Cardioembolic stroke. Continuum (Minneap Minn). (2017) 23 (Cerebrovascular Disease):111–32. doi: 10.1212/CON.0000000000000419 28157747

[3] Rücker V Heuschmann PU O'Flaherty M Weingärtner M Hess M Sedlak C . Twenty-year time trends in long-term case-fatality and recurrence rates after ischemic stroke stratified by etiology. Stroke. (2020) 51:2778–85. doi: 10.1161/STROKEAHA.120.029972, 32811383

[4] Hart RG Diener HC Coutts SB Easton JD Granger CB O'Donnell MJ . Embolic strokes of undetermined source: the case for a new clinical construct. Lancet Neurol. (2014) 13:429–38. doi: 10.1016/S1474-4422(13)70310-7, 24646875

[5] Harris J Yoon J Salem M Selim M Kumar S Lioutas VA. Utility of transthoracic echocardiography in diagnostic evaluation of ischemic stroke. Front Neurol. (2020) 11:103. doi: 10.3389/fneur.2020.00103, 32132971 PMC7040372

[6] de Bruijn SF Agema WR Lammers GJ van der Wall EE Wolterbeek R Holman ER . Transesophageal echocardiography is superior to transthoracic echocardiography in management of patients of any age with transient ischemic attack or stroke. Stroke. (2006) 37:2531–4. doi: 10.1161/01.STR.0000241064.46659.69, 16946152

[7] Hur J Kim YJ Lee HJ Ha JW Heo JH Choi EY . Cardiac computed tomographic angiography for detection of cardiac sources of embolism in stroke patients. Stroke. (2009) 40:2073–8. doi: 10.1161/STROKEAHA.108.537928, 19372451

[8] Ghozy S Liu M Kobeissi H Mortezaei A Amoukhteh M Abbas AS . Cardiac CT vs echocardiography for Intracardiac Thrombus detection in ischemic stroke: a systematic review and Meta-analysis of 43 studies. Neurology. (2024) 103:e209771. doi: 10.1212/WNL.0000000000209771 39270155

[9] Groeneveld NS Guglielmi V Leeflang MMG Matthijs Boekholdt S Nils Planken R Roos Y . CT angiography vs echocardiography for detection of cardiac thrombi in ischemic stroke: a systematic review and meta-analysis. J Neurol. (2020) 267:1793–801. doi: 10.1007/s00415-020-09766-8, 32140869 PMC7293690

[10] Rinkel LA Guglielmi V Beemsterboer CFP Groeneveld NS Lobé NHJ Boekholdt SM . Diagnostic yield of ECG-gated cardiac CT in theAcute phase of ischemic stroke vsTransthoracic echocardiography. Neurology. (2022) 99:e1456–64. doi: 10.1212/WNL.0000000000200995, 35918169

[11] Giruparajah M Bosch J Vanassche T Mattina K Connolly SJ Pater C . Global survey of the diagnostic evaluation and management of cryptogenic ischemic stroke. Int J Stroke. (2015) 10:1031–6. doi: 10.1111/ijs.12509, 25982709

[12] Lee P Dhillon G Pourafkari M DaBreo D Jaff Z Appireddy R . Non-ECG-gated cardiac CT angiography in acute stroke is feasible and detects sources of embolism. Int J Stroke. (2024) 19:189–98. doi: 10.1177/17474930231193335, 37515467 PMC10811964

[13] Senadeera SC Palmer DG Keenan R Beharry J Yuh Lim J Hurrell MA . Left atrial appendage Thrombus detected during Hyperacute stroke imaging is associated with atrial fibrillation. Stroke. (2020) 51:3760–4. doi: 10.1161/STROKEAHA.120.030258, 33161849

[14] Guglielmi V Planken RN Mihl C Niesen S Staals J Coutinho JM . Non-gated cardiac CT angiography for detection of cardio-aortic sources of embolism in the acute phase of ischaemic stroke. J Neurol Neurosurg Psychiatry. (2020) 91:442–3. doi: 10.1136/jnnp-2019-321923, 31974129

[15] Bernard A Leclercq T Comby PO Duloquin G Ricolfi F Béjot Y . High rate of cardiac thrombus diagnosed by adding cardiac imaging in acute stroke computed tomography protocol. Int J Stroke. (2021) 16:692–700. doi: 10.1177/1747493020967623, 33143553

[16] Tomari S Chew BLA Soans B Ai-Hadethi S Ottavi T Lillicrap T . Role of cardiac computed tomography in hyperacute stroke assessment. J Stroke Cerebrovasc Dis. (2024) 33:107470. doi: 10.1016/j.jstrokecerebrovasdis.2023.107470, 38029458

[17] Yu S Zhang H Li H. Cardiac computed tomography versus transesophageal echocardiography for the detection of left atrial appendage Thrombus: a systemic review and meta-analysis. J Am Heart Assoc. (2021) 10:e022505. doi: 10.1161/JAHA.121.022505, 34796743 PMC9075398

[18] Sposato LA Ayan D Ahmed M Fridman S Mandzia JL Elrayes M . Extended CT angiography versus standard CT angiography for the detection of cardioaortic thrombus in patients with ischaemic stroke and transient ischaemic attack (DAYLIGHT): a prospective, randomised, open-label, blinded end-point trial. Lancet Neurol. (2025) 24:489–99. doi: 10.1016/S1474-4422(25)00111-5, 40409313

[19] Amarenco P Cohen A Hommel M Moulin T Leys D Bousser MG. Atherosclerotic disease of the aortic arch as a risk factor for recurrent ischemic stroke. N Engl J Med. (1996) 334:1216–21. doi: 10.1056/NEJM199605093341902 8606716

[20] Lip GY Ponikowski P Andreotti F Anker SD Filippatos G Homma S . Thrombo-embolism and antithrombotic therapy for heart failure in sinus rhythm. A joint consensus document from the ESC heart failure association and the ESC working group on thrombosis. Eur J Heart Fail. (2012) 14:681–95. doi: 10.1093/eurjhf/hfs073, 22611046

[21] Levine GN McEvoy JW Fang JC Ibeh C McCarthy CP Misra A . Management of Patients at risk for and with left ventricular Thrombus: a scientific statement from the American Heart Association. Circulation. (2022) 146:e205–23. doi: 10.1161/CIR.0000000000001092, 36106537

[22] Adams HP Jr Bendixen BH Kappelle LJ Biller J Love BB Gordon DL . Classification of subtype of acute ischemic stroke. Definitions for use in a multicenter clinical trial. TOAST. Trial of org 10172 in acute stroke treatment. Stroke. (1993) 24:35–41. doi: 10.1161/01.STR.24.1.35, 7678184

[23] Yeo LLL Holmin S Andersson T Lundström E Gopinathan A Lim EL . Nongated cardiac computed tomographic angiograms for detection of embolic sources in acute ischemic stroke. Stroke. (2017) 48:1256–61. doi: 10.1161/STROKEAHA.117.016903, 28386043

[24] Kauw F Velthuis BK Takx RAP Guglielmo M Cramer MJ van Ommen F . Detection of Cardioembolic sources with nongated cardiac computed tomography angiography in acute stroke: results from the ENCLOSE study. Stroke. (2023) 54:821–30. doi: 10.1161/STROKEAHA.122.041018, 36779342 PMC9951793

[25] Freedman B Kamel H Van Gelder IC Schnabel RB. Atrial fibrillation: villain or bystander in vascular brain injury. Eur Heart J Suppl. (2020) 22:M51–m9. doi: 10.1093/eurheartj/suaa166, 33664640 PMC7916423

[26] Leung M van Rosendael PJ van der Bijl P Regeer MV van Wijngaarden SE Leung DY . The value of serial echocardiography in risk assessment of patients with paroxysmal atrial fibrillation. Int J Cardiovasc Imaging. (2024) 40:499–508. doi: 10.1007/s10554-023-03014-6, 38148375

[27] Benjamin EJ D'Agostino RB Belanger AJ Wolf PA Levy D. Left atrial size and the risk of stroke and death. The Framingham Heart Study. Circulation. (1995) 92:835–41. doi: 10.1161/01.CIR.92.4.835 7641364

[28] Austein F Eden M Engel J Lebenatus A Larsen N Both M . Practicability and diagnostic yield of one-stop stroke CT with delayed-phase cardiac CT in detecting major Cardioembolic sources of acute ischemic stroke: a proof of concept study. Clin Neuroradiol. (2021) 31:911–20. doi: 10.1007/s00062-021-01003-7, 33688981 PMC8648696

[29] Popkirov S Schlegel U Weber W Kleffner I Altenbernd J. Cardiac imaging within emergency CT angiography for acute stroke can detect atrial clots. Front Neurol. (2019) 10:349. doi: 10.3389/fneur.2019.00349, 31024438 PMC6467937

[30] Khatri RB Assefa Y. Access to health services among culturally and linguistically diverse populations in the Australian universal health care system: issues and challenges. BMC Public Health. (2022) 22:880. doi: 10.1186/s12889-022-13256-z, 35505307 PMC9063872

[31] South Western Sydney Local Health District SWSPHN. South West Sydney: Our Health in Brief. Sydney: NSW Government (2019). p. 31.

[32] Desjardins B Kazerooni EA. ECG-gated cardiac CT. AJR Am J Roentgenol. (2004) 182:993–1010. doi: 10.2214/ajr.182.4.1820993, 15039178

[33] Romero J Cao JJ Garcia MJ Taub CC. Cardiac imaging for assessment of left atrial appendage stasis and thrombosis. Nat Rev Cardiol. (2014) 11:470–80. doi: 10.1038/nrcardio.2014.77, 24913058

[34] Hur J Kim YJ Lee HJ Ha JW Heo JH Choi EY . Left atrial appendage thrombi in stroke patients: detection with two-phase cardiac CT angiography versus transesophageal echocardiography. Radiology. (2009) 251:683–90. doi: 10.1148/radiol.2513090794, 19366905

[35] Spagnolo P Giglio M Di Marco D Cannaò PM Agricola E Della Bella PE . Diagnosis of left atrial appendage thrombus in patients with atrial fibrillation: delayed contrast-enhanced cardiac CT. Eur Radiol. (2021) 31:1236–44. doi: 10.1007/s00330-020-07172-2, 32886202 PMC7880950

[36] Trattner S Halliburton S Thompson CM Xu Y Chelliah A Jambawalikar SR . Cardiac-specific conversion factors to estimate radiation effective dose from dose-length product in computed tomography. JACC Cardiovasc Imaging. (2018) 11:64–74. doi: 10.1016/j.jcmg.2017.06.006, 28823748 PMC5756125

[37] McCollough CH Bushberg JT Fletcher JG Eckel LJ. Answers to common questions about the use and safety of CT scans. Mayo Clin Proc. (2015) 90:1380–92. doi: 10.1016/j.mayocp.2015.07.011, 26434964

[38] Ferrari E Benhamou M Berthier F Baudouy M. Mobile thrombi of the right heart in pulmonary embolism: delayed disappearance after thrombolytic treatment. Chest. (2005) 127:1051–3. doi: 10.1378/chest.127.3.1051, 15764793

[39] Kitmeridou S Tsiptsios D Tsalkidis D Psatha EA Iliopoulos I Aggelousis N . Intravenous thrombolysis for acute ischemic stroke associated with known left ventricular thrombus: safe or not? Neurol Res Pract. (2022) 4:61. doi: 10.1186/s42466-022-00227-3, 36550521 PMC9773473

[40] Poppe M Magnet I Müller M Janata-Schwatczek K. Thrombolysis of a massive intracardiac thrombus during resuscitation: documentation by transoesophageal echocardiography. BMJ Case Rep. (2021) 14:e239063. doi: 10.1136/bcr-2020-239063, 33541993 PMC7868184

[41] Secchi TL Pille A da Silva MMD Martins SCO Bagur R Sposato LA . Neurologists preferences on basic and advanced cardiac imaging utilization in ischemic stroke patients. Cerebrovasc Dis. (2025) 54:350–5. doi: 10.1159/000539998, 38934136

[42] Pathan F Hecht H Narula J Marwick TH. Roles of transesophageal echocardiography and cardiac computed tomography for evaluation of left atrial Thrombus and associated pathology: a review and critical analysis. JACC Cardiovasc Imaging. (2018) 11:616–27. doi: 10.1016/j.jcmg.2017.12.019, 29622180

[43] Sadanandan S Sherrid MV. Clinical and echocardiographic characteristics of left atrial spontaneous echo contrast in sinus rhythm. J Am Coll Cardiol. (2000) 35:1932–8. doi: 10.1016/S0735-1097(00)00643-4, 10841246

[44] Nio SS Rinkel LA Cramer ON Özata ZB Beemsterboer CFP Guglielmi V . Left atrial appendage opacification on cardiac computed tomography in acute ischemic stroke: the clinical implications of slow-flow. J Am Heart Assoc. (2024) 13:e034106. doi: 10.1161/JAHA.123.034106, 39190561 PMC11646529

[45] Zadeh LA. Fuzzy sets. Inf Control. (1965) 8:338–53. doi: 10.1016/s0019-9958(65)90241-x

[46] Bilotta G Barrile V Bibbò L Meduri GM Versaci M Angiulli G. Enhancing land cover classification: fuzzy similarity approach versus random forest. Symmetry. (2025) 17:929. doi: 10.3390/sym17060929

