# Supplementary figures and images for: Intra-cardiac thrombus detection by electrocardiogram-gated cardiac computed tomography in hyperacute ischemic stroke
Source: Front Neurol. 2026 Jun 24;17:1762455. doi: 10.3389/fneur.2026.1762455 (PMC13345591; doi:10.3389/fneur.2026.1762455)

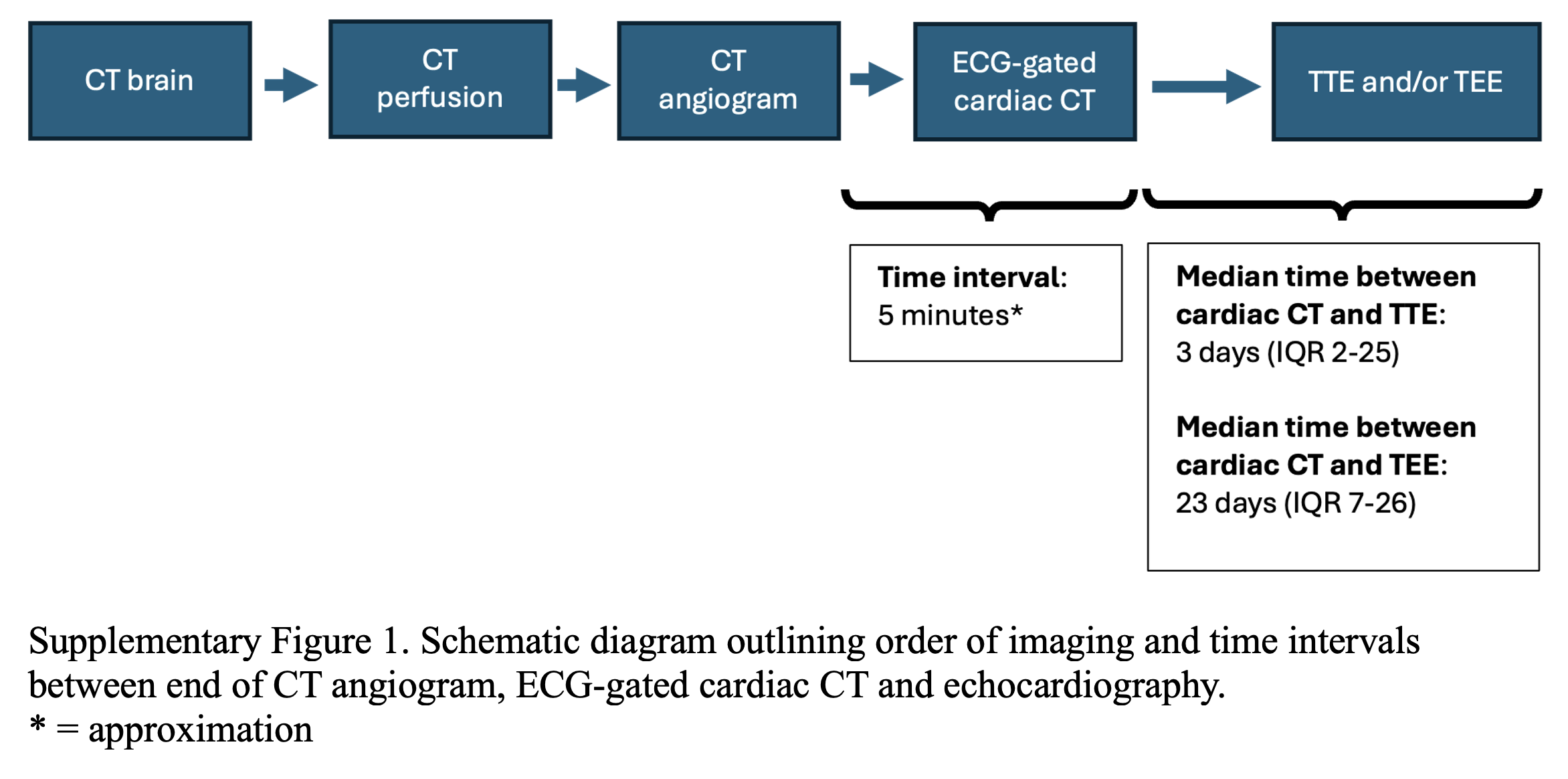

Supplement: SUPPLEMENTARY FIGURE 1 — Schematic diagram outlining order of imaging and time intervals between end of CT angiogram, ECG-gated cardiac CT and echocardiography. * = approximation. [file image_1.png]
